# Nursing Students’ Perceptions and Use of Generative Artificial Intelligence in Nursing Education

**DOI:** 10.3390/nursrep15020068

**Published:** 2025-02-14

**Authors:** ShinHi Han, Hee Sun Kang, Philip Gimber, Sunghyun Lim

**Affiliations:** 1 Health Science/Nursing, LaGuardia Community College, The City University of New York, Long Island City, NY 11101, USA; shan@lagcc.cuny.edu (S.H.); pgimber@lagcc.cuny.edu (P.G.); 2 Red Cross College of Nursing, Chung-Ang University, 84 Heukseok-ro, Dongjak-gu, Seoul 06974, Republic of Korea; goodcare@cau.ac.kr; 3 Columbia University Irving Medical Center, 177 Fort Washington Ave., New York, NY 10032, USA

**Keywords:** use of GenAI, perceptions of GenAI, nursing student, nursing education

## Abstract

**Background/Objectives:** Artificial intelligence (AI) is transforming nursing, with generative AI (GenAI) tools such as ChatGPT offering opportunities to enhance education through personalized learning pathways. This study aimed to explore nursing students’ use of generative artificial intelligence (GenAI) and their perceptions of its use in nursing education, including its advantages, disadvantages, and perceived support needs. **Methods:** This study employed an online survey. The participants were 99 undergraduate nursing students in New York City. Data was collected online through self-report measures using semi-structured, open-ended questions. The data was analyzed using content analysis. **Results:** Most participants (92%) used GenAI tools to access accurate information, clarify nursing concepts, and support clinical tasks such as diagnoses and health assessments, as well as schoolwork, grammar checks, and health promotion. They valued GenAI as a quick, accessible resource that simplified complex information and supported learning through definitions, practice questions, and writing improvements. However, the participants noted drawbacks, such as subscription costs, over-reliance, information overload, and accuracy issues, leading to trust concerns. The participants suggested financial support, early guidance, and instructional modules to better integrate AI into nursing education. **Conclusions:** The results indicate that GenAI positively impacts nursing education and highlight the need for guidelines on critical evaluation. To integrate GenAI effectively, educators should consider introductory sessions, support programs, and a GenAI-friendly environment, promoting responsible AI use and preparing students for its application in nursing education.

## 1. Introduction

Artificial intelligence (AI) is expected to transform every aspect of nursing, including education, clinical practice, administration, and research [1,2,3]. Specifically, incorporating generative AI (GenAI) tools into nursing education through targeted coursework, assignments, training, and experiential learning is anticipated to enhance students’ motivation, engagement, and self-directed learning by providing personalized learning pathways [4,5,6,7,8,9]. Additionally, GenAI-based learning can empower students to enhance their proficiency with AI technology [5]. AI tools can be grouped into two main categories: GenAI and assistive AI. GenAI tools, such as ChatGPT, Just Done, DALL-E, and GitHub Copilot, are designed to generate new content by analyzing and learning patterns from extensive datasets. Assistive AI tools, such as Grammarly, Screen Readers, and Alexa, aim to enhance or support human tasks. Notably, GenAI can also serve assistive purposes, such as providing support for language learning [10].

As AI applications in clinical settings continue to improve care quality and streamline workflows, it is imperative for nursing education to evolve, preparing students for the rapidly changing healthcare environment [3,9,11,12]. Moreover, by integrating GenAI tools into pedagogical practices, nursing educators transition from being mere knowledge facilitators to empowering students by enhancing their sense of autonomy, building their competence, and promoting meaningful interactions with both peers and instructors [10].

Buchanan et al. identified the significant potential of AI in transforming nursing education in both academic institutions and clinical practice, particularly in preparing nursing students for the evolving healthcare landscape [5]. Despite these promising benefits, several challenges remain in addressing ethical concerns, the reliability of information, accuracy, dependency, plagiarism, and breaches of confidentiality in AI technologies [13,14,15,16]. Eacersall emphasized key considerations for the ethical use of GenAI, including the quality of its outputs, data protection, copyright and intellectual property concerns, transparency, and social impacts [17].

Prior research further emphasizes that students’ behavioral intention to use GenAI in their learning is significantly shaped by their attitudes toward AI [14,18]. In line with these findings, Labrague et al. showed that the use of AI tools in nursing education positively impacted students’ perceptions and attitudes toward AI adoption [19]. Similarly, George Pallivathukal et al. identified a positive correlation between healthcare students’ use of ChatGPT and their knowledge and attitudes [13]. However, a study reported that over 60% of nursing students had an unfavorable attitude toward AI, highlighting a gap between its potential benefits and students’ attitudes toward AI [20].

Therefore, exploring nursing students’ experiences and perceptions of GenAI tools is essential to address their integration and develop strategies that effectively incorporate GenAI-based learning into nursing education. Understanding students’ perceptions and usage of GenAI also enables nursing educators to design and adapt curriculum content to better align with their needs and readiness. Additionally, by identifying barriers such as a lack of skills, knowledge, or time, educators can implement strategies to help students overcome these challenges, fostering greater engagement and adoption of AI tools in nursing education [21,22]. Despite the increasing relevance and benefits of GenAI in nursing education, comprehensive studies exploring nursing students’ perceptions and actual usage of these tools remain limited. This study aimed to explore nursing students’ perceptions and use of GenAI in nursing education. The research questions are as follows: What are nursing students’ perceptions of GenAI in the context of their education? How do nursing students utilize GenAI in their learning processes and academic activities?

## 2. Materials and Methods

### 2.1. Study Design

This study employed an online survey with open-ended questions to gain insights into participants’ unique perspectives on GenAI in nursing education.

### 2.2. Participants and Settings

The participants were recruited using convenience sampling and consisted of 99 undergraduate associate nursing degree students from a college in New York City, United States. The inclusion criteria were (1) nursing students enrolled in the Nursing Program for at least one semester, (2) a willingness to participate in research, and (3) aged 18 years or above. The exclusion criteria were students who were not enrolled in the nursing program.

### 2.3. Procedures

Following approval from the Institutional Review Board of the first author’s college and permission from the chair of the Health Science Department, a recruitment flyer was distributed to potential participants enrolled in the RN nursing program. Interested participants accessed an individual Qualtrics link through which data were collected anonymously. Potential participants received an informed consent form through the survey site detailing the study’s purpose, participants’ right to confidentiality, voluntary participation, withdrawal options, and the study’s risks and benefits. Those who provided informed consent were enrolled in the study and completed the questionnaire. No identifying information was collected throughout the online process, thereby safeguarding participant privacy.

### 2.4. Data Collection

Data were collected through online self-report surveys using Qualtrics. Individuals interested in the study were invited to anonymously access the provided Qualtrics link, which featured semi-structured, open-ended questions. The study questions were developed based on prior studies [19,23,24] and refined through team discussions. The following questions were included: (1) Throughout this semester, how often did you use GenAI tools (such as ChatGPT, Just Done, DALL-E, GitHub, and Copilot)? (2) If you used GenAI tools during this semester, why did you use them? (3) What are the advantages of using GenAI tools for learning? (4) What are the disadvantages of GenAI tools? (5) What kind of support do you need to utilize the GenAI tools in nursing education? The survey took approximately 15 min to complete. Data were collected in October 2024. The collected responses were then downloaded in Excel format.

### 2.5. Data Analysis

The data was analyzed using inductive content analysis, as described by Elo and Kyngäs [25]. During the preparation phase, we thoroughly read all the responses to understand the overall sense of the data. In the organization phase, we identified keywords, phrases, and sentences and grouped them into categories through iterative analysis. We then derived themes through abstraction. Finally, in the reporting phase, we presented the study’s findings, including representative examples to illustrate these findings. To verify their findings, the authors independently reviewed the categories and themes against the original data and resolved any disagreements by reaching a consensus.

## 3. Results

### 3.1. Participants’ Characteristics

The study participants were 99 nursing students. The mean age of the participants was 31.26 years (standard deviation [SD] = 7.45, range = 20–54), with 78% of them being women. The majority of the participants (60%) were of Asian/Pacific Islander descent, followed by Hispanic/Latine, White, and African American individuals.

### 3.2. Reasons for Using GenAI and Assistive AI Tools

Most participants (92%) reported using generative AI (GenAI) and/or assistive AI tools at least once during the semester. The participants’ reasons for using GenAI and assistive AI tools are outlined in Table 1. They used those tools to obtain accurate and relevant information; understand concepts and topics related to nursing; clarify information; and search for and apply relevant information in nursing clinical practice, including nursing skills, diagnoses, health assessments, medication interactions, and details about various health conditions.

Additionally, the participants relied on GenAI and assistive AI tools to assist with schoolwork and project tasks, as well as actively engaging in learning, generating, and solving practical problems for studying. They utilized assistive AI tools for writing-related tasks, including checking grammar, rephrasing essays, and improving pronunciation. Finally, they used these tools for health promotion, including planning a healthy diet.

### 3.3. Perceived Advantages of Using GenAI and Assistive AI for Nursing Education

The participants identified several advantages of using GenAI and assistive AI (Table 1). They found them useful as quick resources for obtaining general information, proving knowledge, and generating reliable information. They also recognized these tools as efficient, helpful, and easy to use. Additionally, the participants saw GenAI and assistive AI as a faster way to generate definitions and explain concepts in a simple and helpful manner, aiding their understanding of the various concepts and topics related to the curriculum. Regarding clarification, the participants found GenAI and assistive AI to be beneficial in providing specific information and visual aids and simplifying explanations compared to traditional textbooks. They believed that GenAI and assistive AI could help break down complex information for easier understanding and provide a summary of textbook content. Furthermore, the participants thought that GenAI tools could be helpful in clinical education, including problem-solving and displaying examples of data entry in the DAR (data, action, and response) notes that were not explained in class.

The participants stated that using GenAI and assistive AI helped generate relevant practice questions and verify their accuracy. They perceived that the use of assistive AI improved their writing by enhancing the vocabulary, simplifying information in different languages, and providing references or checking citations. The participants also saw these tools as beneficial for accessing external textbook information, understanding nursing and medical terms, solving mathematical problems with nearly 100% accuracy, and providing fresh ideas.

### 3.4. Perceived Disadvantages of Using GenAI and Assistive AI for Nursing Education

The participants’ perceived disadvantages regarding GenAI and assistive AI are outlined in Table 2. They identified the drawbacks of using GenAI and assistive AI as subscription costs associated with advanced AI tools, the potential to become overly dependent on GenAI and assistive AI, and an overwhelming amount of information and resources that lead to time wastage. The participants were unsatisfied with the limited functionality of GenAI and assistive AI, which failed to comprehend questions properly or provided vague rather than specific responses. Additionally, the participants expressed concerns about accuracy, potential bias, and outdated or incorrect information. There were concerns about potential plagiarism and uncertainty regarding the trustworthiness of the information. One participant remarked, “It (GenAI tool) could provide vague information”, while another stated, “Sometimes it gives incorrect information”.

### 3.5. Perceived Support Needs for Using GenAI and Assistive AI in Nursing Education

The participants stated that obtaining financial support for GPT 4.0 and accessing more information about GenAI tools would be beneficial. The participants expressed a desire to learn how to use GenAI and assistive AI in nursing education. They suggested that having more information on reliable sources, guidance on using and the purpose of using GenAI and assistive AI, and access to learning modules would be helpful. They recommended introducing GenAI and assistive AI tools at the beginning of the semester and providing guidance for using and navigating them on a Blackboard. However, some participants did not favor GenAI and assistive AI, expressing a preference for a good teacher in the learning process. Additionally, other participants were uncertain about the efficacy of GenAI and assistive AI tools, citing a lack of knowledge on how to use them, including tools such as ChatGPT.

## 4. Discussion

This study examined nursing students’ GenAI use and perceptions of its integration into nursing education. Most participants viewed GenAI positively, but some expressed concerns about its limited functions and requested additional support.

### 4.1. Use of GenAI and Assistive AI and Perceived Advantages

The participants in this study primarily utilized GenAI and assistive AI for academic support, such as understanding complex nursing concepts and clinical practice, and they found it helpful in generating new ideas for homework or projects. Additionally, they used assistive AI for writing-related tasks and translating into other languages. In contrast, a study found that 53% of nursing students had prior knowledge about AI, while 37% held a positive attitude toward AI [20]. Similarly to our findings, a survey on the use of ChatGPT by Chinese nursing students reported that students used it for homework and essay writing to enhance their learning experience [12]. Another study with healthcare students in Malaysia also reported that students used ChatGPT to understand concepts and generate questions [13]. This study’s findings and previous studies indicate that individuals are utilizing GenAI and assistive AI to complete their school tasks, and these tools can potentially enhance students’ learning experiences. In addition, nursing students utilized their knowledge in clinical practice to solve nursing problems, apply nursing skills, and conduct patient assessments with the aid of GenAI and assistive tools. These findings, supported by prior studies, underscore the importance of integrating GenAI and assistive tools into nursing education to enhance the development of critical thinking and clinical judgment skills [8,9]. Furthermore, this study highlights nursing students’ positive perceptions of GenAI and assistive AI and their strong intentions to adopt new technologies in their learning experiences, emphasizing the significance of incorporating AI-focused training into nursing education. A study showed that using ChatGPT for academic purposes was associated with a high knowledge level and positive attitudes among healthcare students [13]. This finding supports the idea that providing programs that could enhance nursing students’ knowledge and positively change attitudes would help them use GenAI and assistive AI for scholarly purposes.

### 4.2. Perceived Disadvantages

In a survey, healthcare undergraduate students expressed concerns about data accuracy, plagiarism, and dependency when using ChatGPT for academic purposes [13]. Topaz et al. also addressed the potential challenges of using GenAI for students, including potential plagiarism and the risk of producing misinformation [16]. Furthermore, other researchers expressed concerns about privacy, data security, and the ethical use of GenAI, emphasizing the importance of data protection, transparency, and the consideration of its social impacts [10,17]. In line with previous findings, the participants in this study expressed several concerns, such as plagiarism and the occasional provision of outdated or incorrect information, which could limit its effectiveness. Therefore, strategies must be considered to effectively guide college students in overcoming the potential disadvantages of GenAI and assistive AI use. This includes educating them about its limitations, encouraging critical evaluation, promoting ethical use, and fostering AI literacy. Establishing clear guidelines for AI use in nursing education is essential to ensure responsible and beneficial integration. Van Rensburg and Reedy also strongly advocated for the establishment of clear ethical guidelines by higher educational institutions to promote and uphold academic integrity [10].

In a study conducted in the Philippines, nursing students perceived potential barriers to accessing AI technology, such as a lack of computer skills to navigate AI, a lack of knowledge and awareness, and time constraints [26]. In contrast, the participants in this study found the cost a significant challenge when using GenAI. This indicates that barriers to using AI may vary depending on the individual. This also emphasizes the significance of addressing possible disparities in access to information among students, particularly concerning financial support for AI usage. AI tools currently offer both free and paid versions. The paid version provides more up-to-date content. Therefore, assisting students in accessing the latest version would be beneficial. To better support students’ GenAI use, reducing perceived barriers and increasing access at both the department and institutional levels is essential.

### 4.3. Suggestion for Integration into Nursing Education

The use of GenAI and assistive AI is increasing globally, and there is a growing emphasis on integrating AI into nursing education [5,6]. Nursing educators can utilize AI technology to enhance the student experience [21]. Abujaber et al. emphasized the integration of ChatGPT into nursing education, arguing that it can enhance learning outcomes and students’ educational experiences [23]. Educators should expand their understanding of ChatGPT to improve nursing education outcomes [21]. Furthermore, nursing educators need to recognize the potential of AI technology, such as GenAI, to improve nursing students’ knowledge and ensure academic integrity [22]. While GenAI supports nursing students by enhancing personalized learning, educators must recognize that it cannot substitute the social and emotional dimensions of learning derived from human interactions and teacher–student relationships. A critical responsibility of educators is to strike a balance between leveraging the technological benefits of GenAI and preserving the essential human interactions in nursing education [10]. Therefore, nursing educators need to comprehend students’ perceptions and needs for support in utilizing AI technology in nursing education. In this study, the participants expressed that introducing GenAI at the beginning of the semester or uploading modules to the website would be advantageous. This indicates the importance of including AI usage guidelines during school or course orientation sessions. Considering the positive aspects of integrating GenAI into nursing education, providing education on AI-based active learning would be essential for preparing nursing professionals who confidently utilize GenAI for quality nursing care and contribute to nursing science and evidence-based practice.

A study of Chinese nursing students found that students learned about ChatGPT through the Internet, classmates, teachers, conferences, or lectures [12]. This indicates that students can learn about AI usage through various channels. However, some participants in the study had either never used AI or had negative perceptions of AI use. Incorporating GenAI and assistive AI and their practical applications in nursing education would be beneficial in overcoming this issue. For students with negative perceptions regarding GenAI, implementing a mentor–mentee program with proficient users or providing opportunities to discuss GenAI use among themselves and learn from those with positive experiences could be helpful. Educators can empower students by fostering autonomy and confidence in using AI tools and engaging meaningfully with students [10].

One limitation of this study is the lack of detailed information about the specific types of AI tools that the participants accessed. Future studies should consider addressing this limitation. Additionally, this study has limitations in understanding why some students had negative perceptions. Therefore, future studies are suggested to explore this issue more deeply through interviews.

## 5. Conclusions

This study highlights the positive impact of GenAI and assistive AI on nursing education, with the participants recognizing their value in enhancing learning experiences, supporting academic tasks, and aiding clinical practice. While the participants primarily utilized GenAI and assistive AI for concept clarification, writing assistance, and clinical knowledge, they also noted challenges. These included cost barriers, data accuracy issues, and dependency risks, which underscore the need for guidelines on the ethical use and critical evaluation of AI-generated information. To effectively integrate GenAI and assistive AI into nursing education, educators should consider establishing introductory sessions and support programs at the beginning of the semester. Additionally, they should create an AI-friendly environment that promotes equitable access, student mentorship, and ongoing education on responsible and ethical AI use. Addressing these aspects can help nursing students to harness GenAI and assistive AI tools effectively, preparing them to adopt AI technologies in clinical practice, enhance patient care, and contribute to the advancement of evidence-based nursing. The findings of this study can guide educators in offering targeted training and resources, addressing students’ challenges and recommendations to promote a deeper understanding of AI’s role in nursing education. Nurse educators play an important role in facilitating students’ learning and empowering them to use AI tools responsibly and effectively. Ultimately, this research highlights the importance of equipping future nursing professionals with the confidence and skills to effectively leverage AI in clinical settings, advancing both nursing science and evidence-based practice.

## Figures and Tables

**Table 1:** Participants’ use and perceived advantages of using generative AI (GenAI) and assistive AI.

Theme	Category	Types of AI	Contents
Educational and academic development	Academic tasks	GenAI/ assistive AI	- Homework; - Project.
	Active learning through practice problems	GenAI/ assistive AI	- Generate practice questions to study; - Create practice questions; - Solve questions.
	Writing and language enhancement	Assistive AI	- Check grammar and pronunciation; - Simplify some paragraphs; - Write papers and resume; - Rephrase context/essays; - Citations of study; - Translate languages.
Knowledge acquisition and understanding	Seeking general knowledge	GenAI/ assistive AI	- Use as a search engine for general knowledge.
	Concepts and topics	GenAI/ assistive AI	- Better understanding of difficult concepts; - Receive information about topics related to the course.
	Clarification	GenAI/ assistive AI	- Questions and receive rationales for clarification; - Questions regarding textbooks; - Understand things in detail; - Clarification; - Material explanation.
Clinical practice	Nursing practices	GenAI/ assistive AI	- Nursing skills; - Assessment; - Different diagnoses; - Clinical assignments; - Problem-solving; - How to use DAR (data, action, and response notes.
	Medication information	GenAI/ assistive AI	- Medication; - Drug interactions.
	Medical information seeking	GenAI/ assistive AI	- Medical knowledge; - Explanations about diseases, etc.; - Details for anatomy and physiology; - Information about electrocardiograms and simple verification.
Personal health management	Personal health	GenAI/ assistive AI	- Create high-protein meal plans for the participant and their mother.

**Table 2:** Perceived difficulties/disadvantages related to the use of GenAI and assistive AI.

Themes	Category	Contents
Resources and cost concerns	Cost	- Subscriptions (of advanced AI tools).
	Dependency	- Cannot think independently and depend on AI.
Information quality and reliability	Concerns about wrong information (accuracy)	- Not 100% reliable—not fully trustworthy; - Not all information is accurate; - Some discrepancies in information; - Sometimes, answers do not match the book; - Some not updated; - It is biased.
	Uncertainty	- Unsure of accuracy; - Unsure whether to trust the source; - Uncertain how reliable the sources are; - Unclear under whose credentials it falls.
Cognitive and operational challenges	Information overload	- Too many sources; - Too much information; - Waste of time sometimes.
	Limited function (unsatisfactory)	- Sometimes, it does not understand nursing questions; - Many times, it did not match the context taught; - Limits on certain knowledge like opinions; - Provides vague answers; - Sometimes it provides incomplete answers; not specific.
Ethical integrity	Plagiarism	- Some truths and facts are considered plagiarized.

## Data Availability

The data presented in this study are available on request from the corresponding author due to privacy restrictions regarding participant data.

## Public Involvement Statement

No public involvement in any aspect of this research.

## Guidelines and Standards Statement

This manuscript was drafted against the STROBE (Strengthening the Reporting of Observational Studies in Epidemiology) checklist for cross-sectional research.

## Abbreviation

The following abbreviation is used in this manuscript:

GenAI	generative artificial intelligence.
